# Serendipity

**DOI:** 10.1093/sleepadvances/zpad029

**Published:** 2023-09-20

**Authors:** Meir Kryger

**Affiliations:** Professor Emeritus, Yale University, New Haven, Connecticut, USA

*Serendipity:* The faculty of making happy and unexpected discoveries by accident. Also, the fact or an instance of such a discovery.—Oxford English Dictionary [1].

## Early Years and Medical School

**Figure 1.**
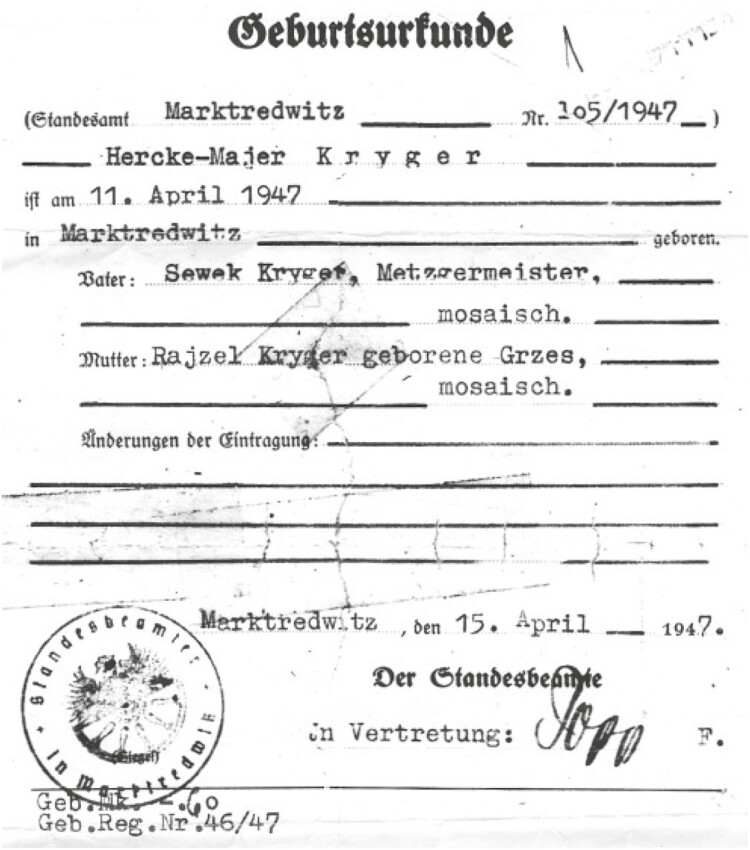
Born a refugee—a person displaced by World War II. This is the birth certificate of the author.

I was the second male born in Germany to a community of Jewish Survivors of the Holocaust in 1947. I discovered the details of how my parents survived the War about forty years after the war ended when my mother for the first time talked about what had happened to her. I learned additional details about how my family survived the War after her story was translated from Yiddish to English and published about fifty years after the War and several years after her death [2, 3]. Our family moved to Israel in 1948 and to Montreal, Canada in 1953. See Figure 1.

All my education was in public schools and later McGill University in Montreal, Canada. I don’t know why I always knew I would become a doctor—no single family member had gone to a university. My father was a butcher who loved to paint; my mother was from a family of bakers. She taught poetry in a slave labor camp during the war. My love of writing and art was likely in my genes.

When I was a young teenager, my father became comatose, and he was taken to the Montreal Neurological Institute. One day when I went to visit him, a doctor was hovering over him with instruments attached to my father’s skull. I asked the doctor what he was doing. He told me he was using radar to try to find out why my father was comatose. Later, the doctor did surgery to evacuate a subdural hematoma and my father woke up. I decided to become a neurosurgeon, just like the doctor, Wilder Penfield. Penfield, I learned much later had described the human homunculus, the functions of regions of the cerebral cortex. This was my first contact with someone I later discovered was a world-famous scientist.

My next contact with Dr. Penfield was while I was a second-year medical student. During his lecture on neurosurgery, he said “If you can’t build a house, don’t go into neurosurgery.” I knew I was clumsy. I changed my career path and decided to focus on the respiratory system. The teachers of respiratory physiology at McGill were enchanting: Joseph Milic-Emili, Peter Macklem, Peter Pare, David Bates, and Nick Anthonisen. I worked in the respiratory labs in the summers where I encountered Larry Wood, Ann Woolcock, and David Flenly. At the time I first encountered these mentors, I had no idea that each was or would become world famous. I was hooked on the control of breathing and physiology [4–6]^.^

I decided to intern in the United States. One day I had lunch with a medical resident in the Royal Victoria cafeteria. That lunch changed the trajectory of my life. Gordon Crelinsten, a year ahead of me found out I was going to intern in Chicago at Michael Reese Hospital. He told me to look up his cousin, Barbara Rosenblum, a student at Northwestern. I did.

## Chicago

### Internship

My Internal Medicine internship was at Michael Reese Hospital in the South Side of Chicago. I wanted to intern in a US hospital to get clinical exposure to conditions I was not likely to see in Montreal. My first choice to intern had been UCSF but I could not be considered because at the time they were not accepting Canadians. That is another story. Decades later I learned that Mark Mahowald (who would become the world’s expert on parasomnias) had been a year ahead of me there, and Stephen Sheldon (who would become one of the world’s best-known pediatric sleep experts) also trained there. On Yom Kippur Eve of 1971, I first met Barbara Rosenblum. She was studying psychology at Northwestern. As an amazing, serendipitous coincidence, a high school friend, Clive Seligman, was working on a Ph.D. in her department. He was the best man at our wedding 3 years later!

Boy, did I learn a lot during internship! I learned to ventilate an asthmatic teenager by hand overnight because the hospital had run out of ventilators. I learned how to sew an ear back on a young girl because the attending was too busy to come to the ER. I learned how to deal with gunshot injuries, arrows piercing the heart, and an icepick severing the spinal cord. I also encountered the first case of a sleep problem in an inpatient.

The patient was an older overweight woman who was being investigated for a troubling symptom of not being able to stay awake. When she was awake, she was completely lucid. We did every test available at the time and could not explain the sleepiness.

## McGill and Patient Zero

The following year I returned to Montreal to complete my internal medicine training at McGill. I also hung out at the Meakins-Christie respiratory research lab. There were two events during this time that would change my life. Both resulted in Grand Round presentations at the Royal Victoria Hospital.

**Figure 2.**
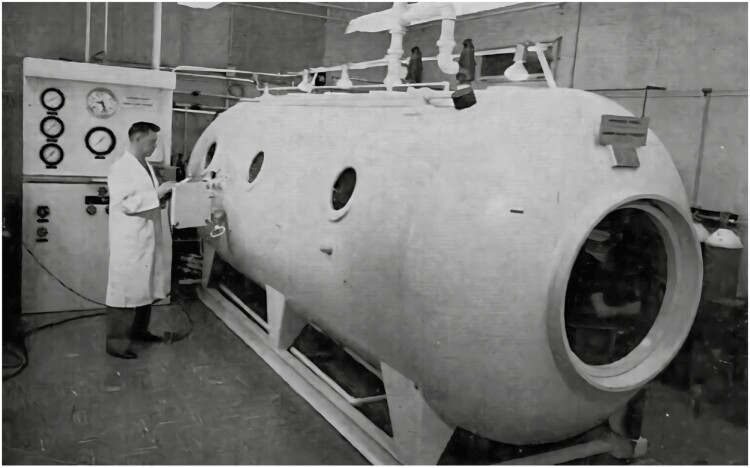
The diving chamber at the Royal Vic.

The first event had to do with the discovery of oil in the depths of the North Sea. There was very little published data about exercise tolerance at extreme depths and my mentor, Dr. Nick Anthonisen who had been a US Navy diver supervised my task which was to do maximal exercise testing in a diving chamber on the third floor of the Royal Vic. After testing a visiting physician from the University of Colorado at a simulated 200-foot depth, we resurfaced using the US Navy 95% diving tables. While walking back to the Meakins with Clifford Zwillich, I suddenly felt left-sided numbness and weakness affecting my leg and arm. I was having a stroke! It was of course the bends. Cliff helped me get back into the chamber, and he and Peter Macklem spent the next 6 frantic sweaty hours with me in the chamber which was repressurized by Nick Anthonisen at the controls outside the chamber. My weakness recovered quickly but the “ascent” to the surface was much slower. I presented myself as the patient at Grand Rounds! This event taught me two things. We had used the 95% tables—I was one of the unlucky 5%—a powerful lesson in statistics. I was already engaged to be married. This event made me realize that research had to be safe for the subject and the researcher. See Figure 2.

The second event started when I was making evening rounds on the endocrine ward on the ninth floor of the Royal Vic. A sleepy obese patient had been admitted with nocturnal seizures accompanied by enuresis. When I went to see the patient, he was asleep and struggling to breathe. I wondered whether this was somehow linked to his seizures. I thought he might have the Pickwickian Syndrome which had been described in 1956. The hypothesis was that the sleepiness in Pickwickian patients was caused by hypercapnia. There were a few reports in obscure European journals of sleep studies in Pickwickian patients and there had been a symposium held in Rimini, Italy on the topic.

**Figure 3.**
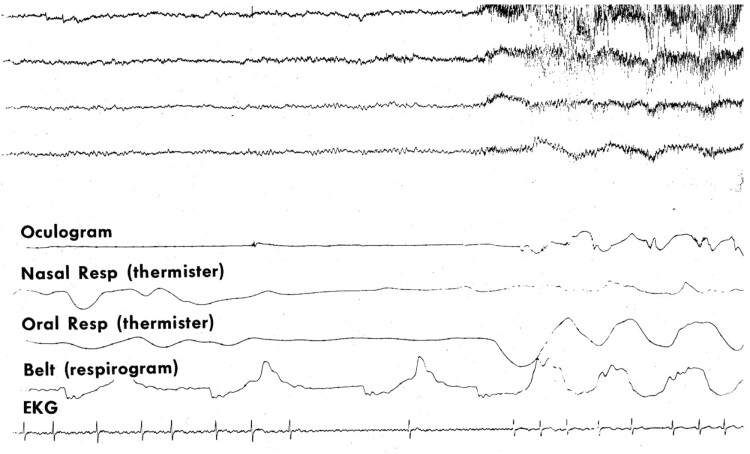
The first respiratory sleep study in Canada in 1973 showed obstructive apnea and asystoles.

My patient was not hypercapnic! He did not fit the literature. A neurosurgical fellow helped set up a primitive polysomnograph that monitored airflow, respiratory effort, EEG, and EKG. This was the first sleep study evaluating a sleep-breathing problem in Canada. We found that the patient stopped breathing due to upper airway obstruction during sleep, resumption of breathing was accompanied by brief awakenings and when he struggled to breathe he developed extreme bradycardia and he had many asystoles, some as long as 10 seconds in duration. We now had an explanation for his seizures—they occurred when his heart stopped. The following morning I went to my attending, who happened to be Nick Anthonisen, and told him that I thought the patient needed a tracheostomy. Nick then said, “Go ahead.” See Figure 3.

The tracheostomy was done and the patient’s sleepiness resolved after one night of sleep and he never had another seizure. I did control of breathing studies on the patient with the tracheal tube plugged and unplugged and confirmed that his drives to breathe were entirely normal, but he had increased upper airway resistance. I wrote up the case and submitted it to the American Journal of Medicine, which had published the original Pickwickian Syndrome article. The paper [7] was rapidly accepted “as is” without any suggested revisions, the only time that happened in my entire publication career (publications by year: Up to the year 2000 [4–139]; since 2000 [140–283]). The patient, of course, had what would eventually be called the Obstructive Sleep Apnea Syndrome, but the term would not be used for several years. I was now hooked on studying the control of breathing, physiology, and sleep! It was serendipity: my interest in control of breathing, and airway obstruction allowed me to understand my patient and that shaped the rest of my career.

## Fellowship

I was now engaged to Barbara. She was accepted to do graduate work in Social Psychology at Colorado State University in Fort Collins. Of course, I had to follow her, so I applied and was accepted for pulmonary fellowship training at the University of Colorado, and married Barbara. At that time there were no medical training matching programs. You wrote letters and got telegrams telling you if you got accepted. Tom Petty (the discoverer of PEEP) was the program director.

My research mentor was John Weil, one of the smartest people I ever met. Although he was trained as a cardiologist, his research was on the control of breathing, especially at high altitudes. Given my interest in sleep, I was to do sleep studies on people with chronic mountain sickness, a condition that was similar to brisket disease in cattle at high altitudes. Up to that point in time nobody had done sleep studies on humans in Colorado. So, I wrote a letter to Ellen Grass (of Grass instruments) requesting sleep recording equipment and she sent me a fully loaded Model 78 polygraph *gratis*.

There was no practical way to measure oxygen levels during sleep in the early 1970s. Serendipitously, the Hewlett Packard company had just developed a big and clunky but beautiful ear oximeter. It now became possible to continuously monitor blood oxygen levels—this was needed for sleep studies at altitude.

A brilliant programmer, Gene McCullough, who had worked for NASA interfaced the polygraph with a microcomputer system so that the massive amount of sleep data could be managed. We set up shop in the hospital in Leadville, the highest permanent habitation in North America. I stayed in Leadville for weeks on end. For our first anniversary, Barbara came to Leadville and helped while I drew blood from my subjects and then we went out for a splendid meal. We did a series of studies that showed that the patients had severe oxygen desaturation during sleep and that respiratory stimulation helped [16–19].

The evolving serendipitous intersection of my interests, physiology, the upper airway, and sleep then led me to write an article called “Diagnosis of obstruction of the upper and central airways” which described the clinical features and flow-volume tracing of patients with upper airway obstruction. At that time, doctors would send letters or postcards to the corresponding author of an article requesting reprints that were ordered from the journal. They were not cheap. I was called into the pulmonary office and informed that they could not afford to handle the reprint requests. There had been over 1500!

The Pulmonary faculty in Colorado was really strong in clinical medicine and cell biology but weak in physiology. So, I was assigned to organize a year-long course on physiology, which I did. I organized an annual Aspen Conference on the control of breathing. I invited well-known scientists including Jerry Dempsey and John Severinghaus and a Frenchman from Stanford University. During breaks in the conference, the Frenchman, Christian Guilleminault, a neuropsychiatrist doing sleep research and I walked in meadows in view of the Maroon Bells. Up to that time, my interest in sleep was confined to breathing and Guilleminault opened my eyes to the breadth of the field. He convinced me to attend my first sleep meeting which was to be held at Stanford. As it turned out I was the only pulmonary person at the meeting—the others were psychiatrists and neurologists. It was then that I was introduced to Bill Dement. He was familiar with my paltry publications and said to me: “My God, you’re a kid.” That was the beginning of a lifelong friendship.

## University of Manitoba

Barbara and I decided to move to Canada. Living in Colorado for 3 years had been great fun—the skiing, the scenery, and the education. We had a view of Long’s Peak from the living room of our townhouse in Longmont. I had been productive in research [13–22] but it was time for a change. The US was in a post-Vietnam War funk. McGill was not a real option—the politics of language was oppressive. I sent letters to these universities: Toronto, Calgary, British Columbia, and Manitoba. Toronto never responded to my letter. I interviewed at the others, but we finally decided on Winnipeg. My old mentor Nick Anthonisen had moved there to be chief of pulmonary, and it had the strongest pulmonary division in the country. The salary was $36,000 a year—it was enough for us. It was 1977. We planned to stay for 4 years. We stayed for 29 years!

**Figure 4.**
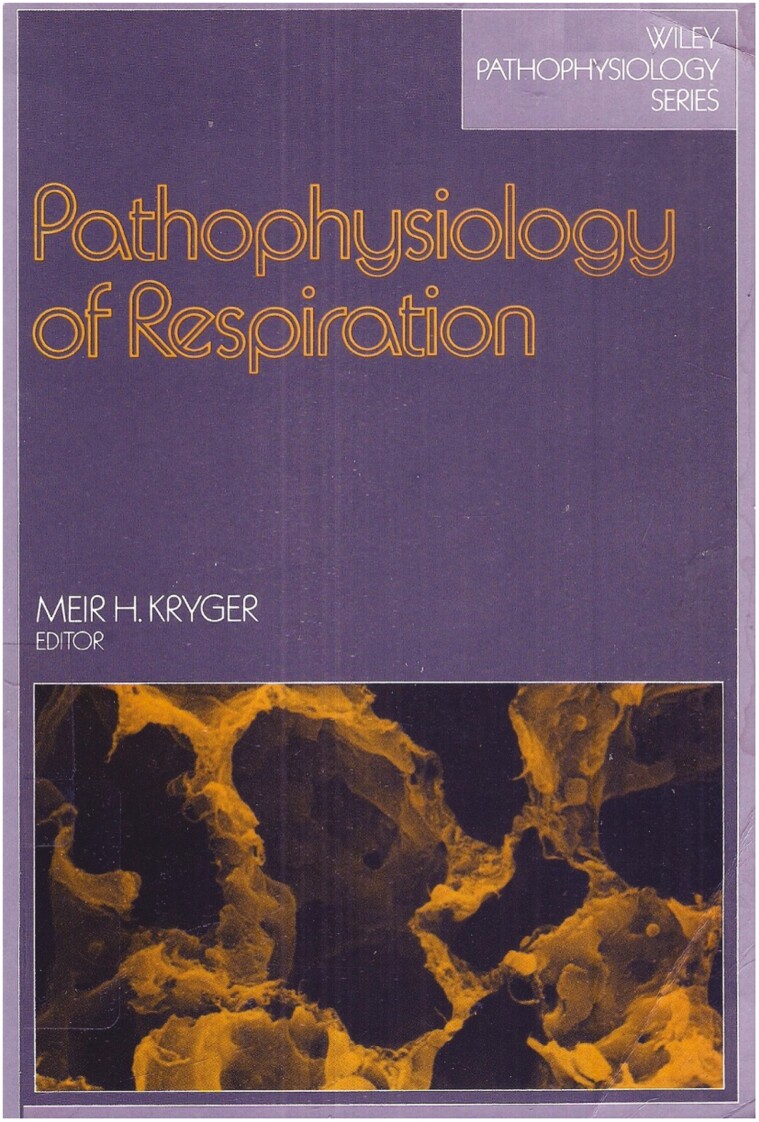
First textbook.

Nick asked me what I wanted. I said that I wanted a sleep lab. I was assigned to St. Boniface Hospital, given an office, a secretary, and space for a tiny sleep lab, and told to start applying for grants. While awaiting grants and equipment I received a letter from the publisher, Wiley, asking me to put together a book on respiratory physiology. They didn’t realize I was a kid. I had nothing else to do, so I put together my first book, *Pathophysiology of Respiration* [30]. I realized that I loved to write. See Figure 4.

Funding and equipment finally arrived. I was able to hire staff and a computer programmer. From my experience as a fellow in Colorado, I knew that it was going to become important to marry the polygraph with a computer system for proper data analysis. The system would need an analog-to-digital converter and a system that could store and process streams of data. This was in 1977. The Apple computer company which was born the year before did not have any products that could be used. The PC did not exist yet. The system we settled on was the Digital Equipment Micro PDP-11/23 which had 512 KB of RAM, a 5 MB hard drive, and two large floppy drives. It cost about $25,000 (a third of the cost of our first house in Winnipeg) and was big and clunky but it worked! [44] It may have been the first computer system to acquire and process and analyze respiratory data during sleep.

Training the staff to be sleep techs was a challenge. There were no courses available so I had to teach them, and whenever a new trainee arrived they needed to be taught. I learned how to program with an environment called Toolbook and I put together a comprehensive program called *Journey into Sleep*. Toolbook was developed by Paul Allen, one of the founders of Microsoft, and at times when I needed tech support I communicated with him. The program was used in many institutions, including as I discovered decades later, my final academic home, Yale.

**Figure 5.**
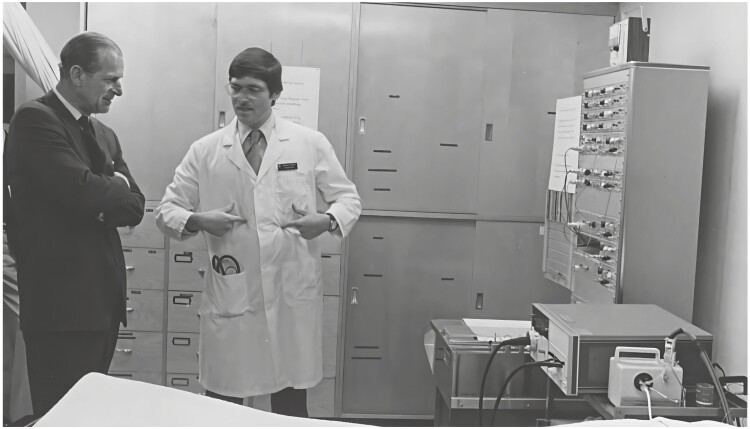
Prince Philip is shown how a sleep study is done in 1978. Note the Grass Model 78 and the HP Oximeter.

Over my 29 years in Winnipeg, I was busy clinically and I mentored trainees from all over: Australia, China, France, Greece, Israel, Japan, Saudi Arabia, Syria, Switzerland, and Canada. Several of the trainees became prominent scientists in their home institutions. Sleep was a new field and there was so much to discover, learn, and teach. There was a great deal to learn about heart failure, sleep-breathing disorders including patients with COPD, restrictive lung diseases, neurological disorders, movement disorders, endocrine disorders, sleep in normal people, and healthcare utilization [2, 23–29, 31–189]. The clinic and lab became well-known internationally—Prince Philip came for a tour of the lab. The lab was a site for many clinical trials of medications and devices. See Figure 5

In 1988, Jiang He, a doctor from China arrived at my office and told me he had been given a fellowship position in my lab. It was a complete surprise to me. I was not expecting him. He told me he was pretty sure about that. He was good at statistics, so I found a great project for him. Tom Roth at Henry Ford Hospital in Detroit had long-term outcome data for a large group of untreated apnea patients. Continuous positive airway pressure (CPAP) had been introduced 3 years before, so data from a large number of patients was available for analysis. After the data were analyzed and showed that patients with an apnea index greater than 20 had increased mortality, the manuscript was submitted to the New England Journal of Medicine. It was instantly rejected without being reviewed; the editor wrote that the topic was of little interest to its readers. It was resubmitted to Chest and quickly accepted and it became the most widely cited paper in sleep medicine for several years [70]. After the paper was published Dr. He told me that there had been a big mistake and he had been directed to the wrong lab when he arrived. He was supposed to be at a lab studying the physiology of the lower airway, not the upper airway. So just as quickly as he had arrived, he disappeared.

For about my first 13 years in Winnipeg, I was attending in chest clinic and the ICU and of course, saw almost every type of organ failure and patients with that mysterious illness caused by HIV. During those years there was no funding to do clinical sleep studies. The medical society and government people were not interested. They could not believe that sleep posed any sort of danger. During that time, I studied thousands of patients with my equipment when it was not needed for research. The sleep clinic and program received official funding in about 1990. By the time I left Winnipeg in 2006, I had studied and managed almost 30,000 people with sleep disorders! When I started, I was the only doctor with expertise in sleep for a vast area—Manitoba, Saskatchewan, Western Ontario, and the Canadian Arctic. Many patients were Indigenous people. It was heartbreaking to learn from my patients about the injustices that they had and continued to endure. In a novel I wrote, *The Man Who Couldn’t Stay Awake*, one of the most important characters is an Indigenous person.

During those years in Winnipeg, my family grew—Shelley, Michael, and Steven arrived. Barbara and they were often the audiences of talks I was rehearsing. They heard them so often, I think they could have given them.

## Yale

Toward the end of my years in Winnipeg, I felt that after 29 years it was time for a change. My three children were all at American Universities. It was time to move. New England was a logical location. I turned down a position at Harvard—we simply could not afford to live in Boston. Yale did not have a position available. So, I accepted a position as Director of Sleep Medicine Research and Education at Gaylord Hospital. The sleep clinic had been originally founded by Yale faculty and was then by far the biggest in the state. It was an unlikely part of a hospital devoted to rehabilitation. Although I was successful in securing research funding, the fit wasn’t quite right. I was an academic and the place simply wasn’t academic enough for me.

A position opened up for me at Yale. It was gutsy for Yale to hire me at my age. The position fit me like a glove! I became the sleep medicine fellowship director. Although there was an existing fellowship program with one position and excellent faculty, it had elements of being *ad hoc*. It did not have secure funding. With Naftali Kaminski’s support, I was able to secure funding for four slots. A comprehensive educational program was established with weekly rounds which eventually had ties with rounds at other institutions around the country.

At first, I was half-time at the VA. I started a remote CPAP monitoring program, one of the first at a VA. I started a program for the treatment of veterans with sleep apnea with oral appliances, also one of the first at a VA.

Perhaps the highlight of my career at Yale was starting an undergraduate course, *The Mystery of Sleep.* In the first couple of years, there were about 20 students. By 2023 there were 85 students, and we had to turn away more than 100 because there were no available classrooms big enough. During the coronavirus disease (COVID) pandemic the course was done remotely. Yale students from Europe, North America, Asia, and Australia attended these remote lectures.

## Giving Back

Bill Dement encouraged me to get involved in an educational capacity with the American Sleep Disorders Association (later called the American Academy of Sleep Medicine). After a meeting in Banff, in the early 1980s, I was given a ride back to the Calgary airport by Carol and Philip Westbrook. We decided that it was time for the organization to get into the education business and that a National Sleep Medicine course was needed. We put together the very first course. It was held in Arizona and I had the honor of inviting and getting to know many of the luminaries in sleep. The course then moved to Leesburg, Virginia where classes were held for several years. At about the same time educational materials were developed, such as slide sets on various topics. I was a member of the board of directors for several years and eventually became president. I had the honor of writing the first article in the first issue of the Journal *SLEEP*.

At about the same time, I was also active in the Canadian Sleep Society and became its president. I helped organize two large international meetings, one in Montreal and one in Quebec City. The latter was a meeting of the World Sleep Apnea Association held on the 25th anniversary of the description of CPAP by Colin Sullivan who gave the keynote; there were over a thousand attendees.

**Figure 6.**
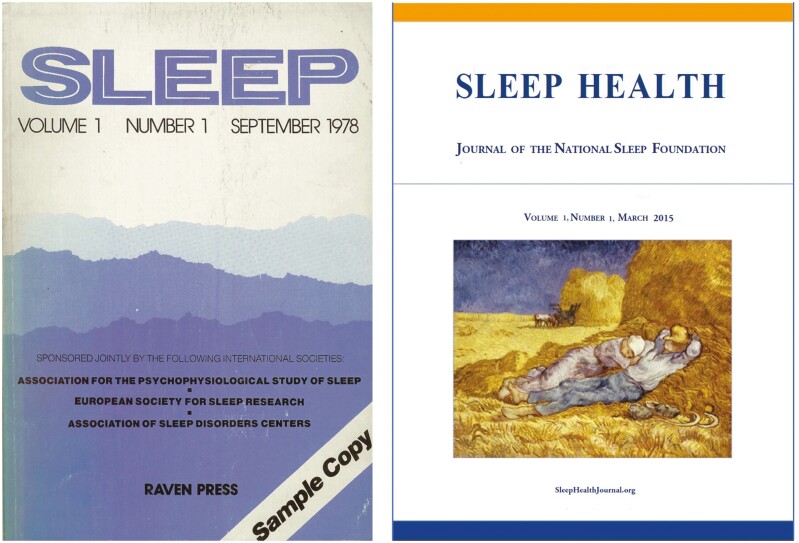
First article in *SLEEP*, 1978;. First article in *SLEEP HEALTH*, 2005.

When I finally rotated off the AASM board, Tom Roth asked me to join the board of the National Sleep Foundation and eventually was elected its chair. Again, my focus was education. I organized several symposia including sleep in older people, sleep in women, sleep and obesity, and sleep and pain. Not long after I rotated off the foundation board it launched a new journal *Sleep Health*. I was asked to be its art editor with the task of picking sleep-themed works of art for its covers and writing about them. This allowed me to continue to explore my lifelong passion for art, and at times communicate with living artists and interact with them. See Figure 6.

These organizations had the most eminent, brightest, and most influential people in sleep medicine. Their backgrounds ranged from the sciences, law, government, and industry. Many became lifelong colleagues and friends. When it came to organizing books, I knew whom to contact.

## Books

Barbara and I spent a sabbatical year in Geneva, Switzerland where I helped set up the first clinical sleep lab in Switzerland. We created a clone of our data acquisition system in Winnipeg. During that year, it was 1983, I received a letter that would change the direction of my life. The letter from an acquisitions editor at Saunders asking if I would be interested in putting together a volume of *Clinics in Chest Medicine* on sleep. I agreed and I asked fellows I had trained and colleagues with whom I had worked to contribute. The first chapter was called *Fat, Sleep and Charles Dickens*. Work on the chapter ignited my interest in the history of sleep medicine. The success was noted by senior editors at the publishing firm.

In about 1984 I was invited to be a speaker by the California Thoracic Society at a meeting held at The Ahwahnee Lodge in Yosemite Park. There, I first met Tom Roth. This was the beginning of a beautiful lifelong friendship. At breakfasts and riding on chairlifts in the beautiful park, we learned about each other and how World War II had impacted our lives. We also realized that the science of sleep was still in its infancy and a textbook was needed. Tom knew everybody in sleep research; I knew everybody in respiratory sleep medicine.

A letter from a senior editor at Saunders asked me if I would be interested in compiling a comprehensive textbook. I was not sure.

**Figure 7.**
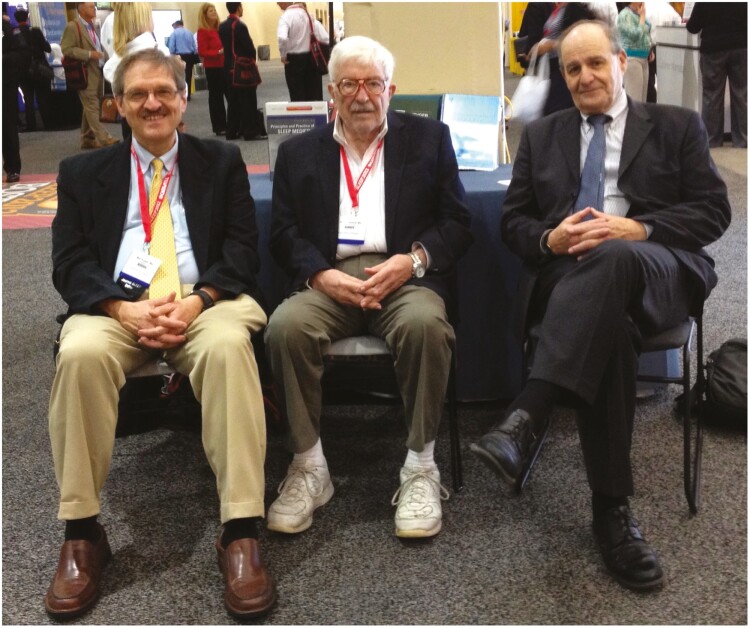
The three amigos worked together for over 30 years. From right to left: Tom Roth, Bill Dement, Meir Kryger.

In about 1985, I was asked by Bill Dement to come to Stanford University and review one of their big grant applications. On that visit, I spoke to Christian Guilleminault about the textbook Tom and I were thinking about. I wanted him to be a senior editor. Astonishingly, Christian was not interested. He told me “there was not enough meat for a book.” I then spoke to Bill Dement. He told me, “You can’t have a medical field without a textbook.” Game on! The task for the next 40 years was set. See Figure 7

**Figure 8.**
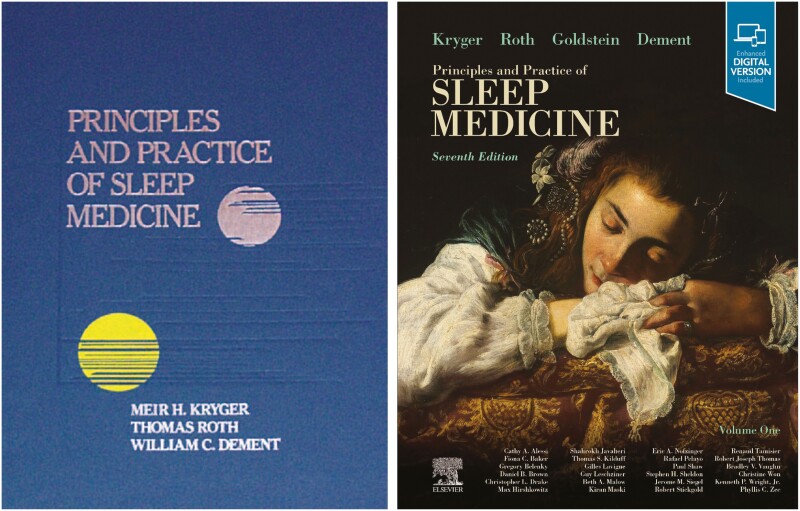
The first edition of Principles and Practice of Sleep Medicine was published in 1989.; The seventh edition in 2022.

The field of sleep medicine was still tiny. Between Bill, Tom, and me, we knew almost everybody involved in sleep medicine around the world. For me, it was a great honor to communicate with the most brilliant minds in sleep research. We found academics who would be section editors who would then find chapter contributors. This era was before the word processor was established, and all the manuscripts, polysomnogram traces and figures were sent back and forth by regular mail. I had to learn all about the publication process. I was involved in the design of the interior of the book and its cover. The first edition of *Principles and Practice of Sleep Medicine* came out in 1989. Cathy Goldstein joined the senior editorial team as we were preparing for the seventh edition. This edition had a difficult gestation because COVID swept over the planet. Some contributors were stricken with the infection, and some were isolated at home and couldn’t access their offices. Some authors died. Some authors just disappeared. My beloved friend and co-editor, Bill Dement died. Cathy Goldstein who had amazing energy and editorial skills played a key role in helping Tom and me complete that edition. It came out in early 2022, during the height of the Omicron wave. See Figure 8.

Then came other books for doctors.

Over the decades when I had patients with interesting findings or stories, with their written permission I would digitally document them, expecting one day to produce a visual atlas. I suggested this to my editors at Elsevier. They liked the idea. The *Atlas of Clinical Sleep Medicine* came out in 2010. The third edition came out in 2023. The first *Sleep Medicine Review* book came out in 2011. Two other editions followed. I was a coeditor of pediatric sleep medicine textbooks.

And then came books for the public.

I did a Canadian national radio interview with a patient about narcolepsy. Twenty minutes after the interview ended, I received a call from a literary agent in Toronto who suggested a book on how sleep disorders impact women. *Can’t Sleep, Can’t Stay Awake* was published in 2004. *Mystery of Sleep* was published in 2017.

Over 30 years ago I wrote a novel as a vehicle that highlighted my thoughts about what I had learned from my Indigenous patients. It resided on hard drives on old computers for three decades. I never really did anything with it until COVID, when I thought if not now, when? The *Man Who Couldn’t Stay Awake* was finally published.

My lifelong fascination with art and sleep culminated in the book, *Sleep in Art,* a volume I originally conceived for the undergraduate class I was teaching at Yale, *Mystery of Sleep.*

As homage to my parents, who were Holocaust survivors, I re-edited and published *Rose’s Odyssey*. My birth is on page 199.

## The one less traveled

I shall be telling this with a sigh

Somewhere ages and ages hence:

Two roads diverged in a wood, and I—

I took the one less traveled by,

And that has made all the difference.

- Robert Frost, from *The Road Not Taken,* 1915

Serendipity played an important part in my life’s journey. The chance encounters with giants of science and medicine and the chance intersection of developing interests and time happened over and over again. The most important chance encounter was in 1970 in the Royal Victoria Hospital when the upper-class medical student told me to look up his cousin, Barbara Rosenblum who was a student at Northwestern. I did, and it changed my life.
